# pH-Responsive Drug Delivery Nanoplatforms as Smart Carriers of Unsymmetrical Bisacridines for Targeted Cancer Therapy

**DOI:** 10.3390/pharmaceutics15010201

**Published:** 2023-01-06

**Authors:** Joanna Pilch, Agnieszka Potęga, Agata Kowalczyk, Artur Kasprzak, Patrycja Kowalik, Piotr Bujak, Ewa Paluszkiewicz, Ewa Augustin, Anna M. Nowicka

**Affiliations:** 1 Faculty of Chemistry, Gdańsk University of Technology, Narutowicza Street 11/12, 80-233 Gdańsk, Poland; 2 Faculty of Chemistry, University of Warsaw, Pasteura Street 1, 02-093 Warsaw, Poland; 3 Faculty of Chemistry, Warsaw University of Technology, Noakowskiego Street 3, 00-664 Warsaw, Poland

**Keywords:** unsymmetrical bisacridines, folic acid, targeted cancer therapy, quantum dots, drug delivery nanosystem, drug release, lysosomal content

## Abstract

Selective therapy and controlled drug release at an intracellular level remain key challenges for effective cancer treatment. Here, we employed folic acid (FA) as a self-navigating molecule in nanoconjugates containing quantum dots (QDs) and *β*-cyclodextrin (*β*-CD) for the delivery of antitumor unsymmetrical bisacridine compound (C-2028) to lung and prostate cancers as well as normal cells. The bisacridine derivative can form the inclusion complex with *β*-cyclodextrin molecule, due to the presence of a planar fragment in its structure. The stability of such a complex is pH-dependent. The drug release profile at different pH values and the mechanism of C-2028 release from QDs-*β*-CD-FA nanoconjugates were investigated. Next, the intracellular fate of compounds and their influence on lysosomal content in the cells were also studied. Confocal Laser Scanning Microscopy studies proved that all investigated compounds were delivered to acidic organelles, the pH of which promoted an increased release of C-2028 from its nanoconjugates. Since the pH in normal cells is higher than in cancer cells, the release of C-2028 from its nanoconjugates is decreased in these cells. Additionally, we obtained the concentration profiles of C-2028 in the selected cells treated with unbound C-2028 or nanoconjugate by the HPLC analysis.

## 1. Introduction

Despite significant progress in cancer therapy, neoplastic diseases are the second leading cause of mortality in the world [1]. Several methods of cancer treatment have been developed, including chemotherapy, radiotherapy, surgery, and immunotherapy [2]. Nevertheless, the most common among these methods remains chemotherapy [3]. Effective usage of common drugs in chemotherapy is often restricted due to their poor solubility, low biodistribution, systemic circulation as well as aggregation [4]. In addition, the non-specific delivery of these drugs is often associated with the use of higher doses to achieve the therapeutic dose, which can induce toxic effects on normal cells [5]. Therefore, it is desirable to develop an effective method of drug delivery, including their selectivity toward cancer cells and reducing adverse side effects together with improving therapeutic efficacy. In recent years a better understanding of cancer biology and increased availability of materials such as nanoparticles (NPs), has led to the development of delivery platforms of chemotherapeutics. The most popular types of NPs for cancer therapy include micelles, liposomes, dendrimers, metallic, and polymeric NPs, as well as quantum dots (QDs) [6,7,8,9,10]. Furthermore, these drug delivery platforms can be modified e.g., by conjugation to antibodies, enzymes, peptides, or targeting cell-surface proteins whose expression is often enhanced on the membrane of cancer cells [11]. Many receptors are known to be overexpressed in cancer cells, like folic acid receptor (FR) [12], transferrin receptor (TfR) [13], or epidermal growth factor receptor (EGFR) [14], which have been explored as docking sites for selective targeting of anticancer chemotherapeutics [15]. Therefore, these drugs can be selectively delivered to cancer cells and tissues by conjugating drugs and NPs with ligands against these receptors. Currently, FRs and TfRs, due to the high affinity of FA (folic acid) and Tf (transferrin), respectively, have been intensively studied for drug delivery [16,17]. Another crucial aspect of “smart” drug delivery remains the triggered release of drugs from a nanocarrier in response to some stimulus encountered on entry into the diseased cell or tissue e.g., a change in pH [18]. The difficulty of controlled drug release at an intracellular level remains a key challenge for maximizing drug safety and efficacy. When designed appropriately, an in vitro release profile can reveal fundamental information on the dosage form and its behavior as well as provide details on the drug release mechanism and kinetics, enabling a rational and scientific approach to drug product development.

Our previous work presented the application of the quaternary QDs (Ag-In-Zn-S nanocrystals) as a platform for transporting unsymmetrical bisacridines (UAs: C-2028 and C-2045) [19]. UAs are a novel prosperous class of anticancer-active acridines developed in our department for many years and the C-2028 compound is their representative derivative. Although dimeric compounds with pharmacological properties are known from the state of the art, UAs are distinguished from other acridine dimers by an extraordinary (unsymmetrical) structure. These are combinations (by using an appropriate flexible linker) of monomeric imidazoacridinones and 1-nitroacridines, which together confer unique physicochemical and biological properties not observed in the case of single monomers. The high cytotoxic activity of UAs has been revealed in many human cancer cell lines, including pancreatic, lung, prostate, and colon. UAs were also active against numerous tumor xenografts in nude mice (e.g., lung and colorectal cancers) and against Walker 256 adenocarcinoma in rats [20]. The promising results from these initial studies give hope for the application of UAs in the treatment or prevention of tumors that are usually resistant to therapy. Moreover, C-2028 was shown to induce apoptosis in HCT116 and H460 cells and accelerated senescence in H460 cells [19]. To gain maximum benefits from these drug candidates we developed their nanoconjugates containing QDs. Recently, we showed that conjugation of UAs to QDs successfully increased the cytotoxic activity and cellular uptake of these compounds in cancer H460 cells and had protective effects on normal MRC-5 and CCD 841 CoN cells [19,21]. Next, we investigated the potential of FA as a self-navigating molecule in QDs-C-2028 nanoconjugates (QDs-*β*-CD(C-2028)-FA) [22]. Conjugation of FA to nanoparticles significantly increased the amount of delivered anticancer agent, especially to human H460 lung and human Du-145 prostate cancer cells. 

In this paper, as the next step in our research, we report the mechanism of drug release from nanoconjugates. We assessed the influence of pH on the drug release profile. For these studies, we selected the C-2028 compound as a lead compound with the highest cytotoxic and antitumor activity in a group of UAs. We show the degradation pathway and the cellular fate of QDs-*β*-CD(C-2028)-FA nanoconjugates and their influence on the lysosome content in the human lung (H460) and prostate (Du-145 and LNCaP) cancer cells as well as in their corresponding MRC-5 and PNT1A normal cells. In addition to the analyses made at an intracellular level, we also evaluate the release behavior of C-2028 from nanoconjugates by a classical approach using the reversed-phase high-performance liquid chromatography (HPLC) method. HPLC is a standard bioanalysis method for small molecule drugs, which recently is also considered the preferred choice for profiling the drug release process of NPs in vivo.

## 2. Materials and Methods

### 2.1. Materials

1-ethyl-3-(3-dimethylaminopropyl)carbodiimide hydrochloride (EDC × HCl), 4 dimethylaminopyridine (DMAP), dimethyl sulfoxide (DMSO), folic acid (FA), formic acid, penicillin, streptomycin, and *β*-cyclodextrin (*β*-CD) were all purchased from Sigma-Aldrich, St. Louis, MO, USA. LysoTracker Red DND-99 and Alexa Fluor^®^ 647 phalloidin were purchased from Invitrogen, Eugene, Oregon, USA. Methanol (gradient grade quality for HPLC) was obtained from Merck KGaA, Darmstadt, Germany. All used reagents and chemicals were of analytical grade and were used as received without any further purification. The synthesis method of C-2028 compound 9′-{N[(imidazo[4,5,1-de]-acridin-6-on-5-yl)aminopropyl]-N-methylaminopropylamino}-1′-nitroacridine and QDs (QD_green_ and QD_red_) were as previously described [22]. Ultrapure water (conductivity 0.056 μS·cm^−1^) was used for the preparation of all aqueous solutions (Milli-Q^®^ IQ 7005 Water Purification System, Merck KGaA, Germany).

### 2.2. Synthesis of QDs-β-CD(C-2028)-FA Nanoconjugates

**Figure 1:**
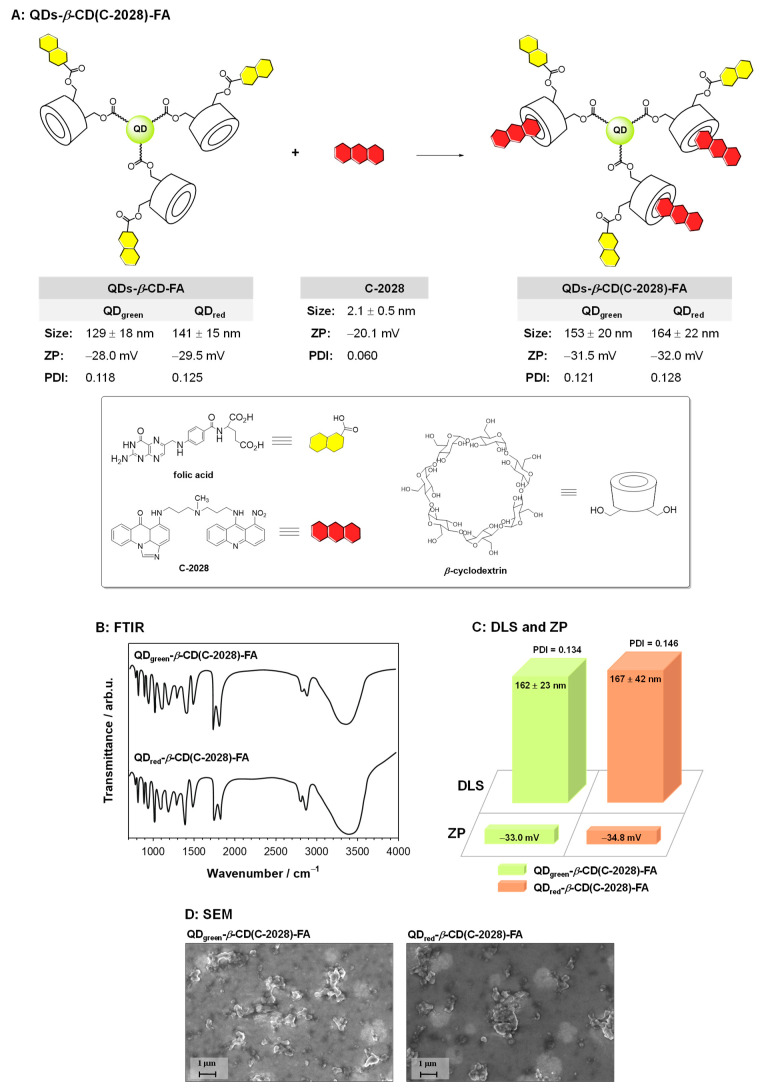
Scheme of QDs-*β*-CD(C-2028)-FA nanoconjugate synthesis and values of the mean hydrodynamic diameter, zeta potential (ZP), and polydispersity index (PDI) were determined in 0.02 M PBS (pH 7.4) (**A**). FTIR spectra (**B**), DLS and ZP (**C**), and SEM images (**D**) of QDs-*β*-CD(C-2028)-FA nanoconjugates.

In the role of nanocarrier, the QDs-*β*-CD-FA hybrids were applied. The core of the hybrid was quantum dots: (i) QD_green_, Ag_1.0_In_1.2_Zn_5.6_S_9.4_ or (ii) QD_red_, Ag_1.0_In_1.0_Zn_1.0_S_3.5_. To facilitate the conjugation process with *β*-cyclodextrin (*β*-CD) the surface of the quantum dots was decorated with 11-mercaptoundecanoic acid (MUA). The procedure of the QDs-MUA preparation was described in the literature [23,24,25]. The introduction of *β*-cyclodextrin molecules to the surface of quantum dots as well as the functionalization of the obtained QDs-*β*-CD nanoconjugate with folic acid was based on the formation of ester bonds. The procedure of QDs-*β*-CD-FA hybrids synthesis is described in detail in our previous paper [22] concerning the physicochemical properties of these hybrids. The unsymmetrical bisacridine derivative (C-2028) was anchored to the QDs-*β*-CD-FA hybrid through the formation of an inclusion complex with *β*-cyclodextrin. The water mixture of the QDs-*β*-CD-FA hybrid (1.0 mg·mL^−1^) and C-2028 (300 µM) was stirred overnight at room temperature (RT) and then dialyzed (five times) against distilled water to remove the unbound unsymmetrical bisacridine derivative. The scheme of the synthesis of the QDs-*β*-CD(C-2028)-FA nanoconjugates as well as the physical properties like hydrodynamic diameter and zeta potential (ZP) is presented in Figure 1. Each time, the synthesized QDs-*β*-CD(C-2028)-FA nanoconjugates were characterized using FTIR spectroscopy and scanning electron microscopy. The synthesis of both QDs-*β*-CD-FA hybrids and QDs-*β*-CD(C-2028)-FA nanoconjugates was repeated three times. The results are shown in Figure 1. At each time, bands characteristic of the individual components of the conjugate were observed on the FTIR spectra, the intensity of which varied slightly. An analogous trend of changes was observed for DLS and ZP measurements. The average hydrodynamic diameter and zeta potential values between the series did not differ more than 15% between each other.

### 2.3. Physicochemical Characteristic of QDs-β-CD(C-2028)-FA Nanoconjugates

The dynamic light scattering (DLS) and zeta potential (ZP) measurements were carried out in 0.02 M phosphate-buffered saline (PBS) pH 7.4 at 21 °C with a Zetasizer nano series apparatus (Malvern Panalytical, Malvern, UK) with a He-Ne (4 mW) laser at 632.8 nm. The UV-Vis spectra were recorded using Lambda 25 spectrometer (PerkinElmer, Waltham, MA, USA), in the range of 200–900 nm at RT in a quartz cuvette of 1 cm length of the optical window. The FTIR spectra were acquired in a transmission mode on Perkin Elmer System 2000 spectrophotometer (Waltham, MA, USA) in the range of 400–4000 cm^−1^ with a spectral resolution of 4 cm^−1^. The pellets were prepared from a mixture of 300 mg of spectrally pure KBr with about 1 wt% of the sample. The QCM-D experiments were carried out with QCM E4 apparatus (Q-sense, Biolin Scientific AB, Gothenburg, Sweden) and with using 4.95 MHz gold quartz crystals. Before the experiments, gold crystals were cleaned according to the TL1 procedure. The crystals were immersed into a mixture heated to 75 °C for 5 min. Then the crystals were rinsed thoroughly with ethanol and distilled water and dried with a stream of argon. For SEM examination, low kV electron beam energy was used (3 kV, 30 pA current). Before the examination, each sample was covered with a 1–2 nm thin film of Au-Pd alloy to avoid electrical charging of the sample surface. The layers of the alloy were sputtered using a Polaron SC7620 Mini Sputter Coater.

### 2.4. C-2028 Release and Its Kinetic Evaluation—In Vitro Studies

The drug release profiles were studied in the non-cellular system using the quartz crystal microbalance with dissipation monitoring (QCM-D) technique. The experiments were performed in PBS at various pH (7.4, 5.2, and 4.0). The mechanism of C-2028 release from QDs-*β*-CD-FA nanoconjugate was fitted by studying its degradation profile with Higuchi and Korsmeyer–Peppas models according to Equations (1) and (2) describing these models, respectively:

(1) $$Q=k_{H}t^{0.5}$$

where *Q* is the percentage of C-2028 released at time *t* and *k_H_* is a release rate constant (Higuchi constant)

(2) $$Q={kt}^{n}$$

where *Q* is the percentage of drug release at time *t*, *k* is a release rate constant, and “*n*” is the release exponent describing the release mechanism [26]. According to the Korsmeyer–Peppas model for *n* ≤ 0.43 drug release kinetics occur according to Fickian diffusion, for 0.43 < *n* < 0.85 the release is described as anomalous, and for *n* ≥ 0.85 drug release is dominated by an erosion mechanism.

QCM-D measurements were carried out with a QEM 404 instrument (Q-Sense, Biolin Scientific, Sweden) equipped with 4.95 MHz AT-cut gold-coated quartz crystals. Before each experiment, the Au crystals were cleaned by immersing them in the heated (75 °C) TL1 solution (ultrapure water, 25% ammonia, and 30% hydrogen peroxide in a volume ratio of 5:1:1) for 5 min. Next, the crystals were rinsed with ultrapure water and dried with Ar stream. The measurements were carried out in the flow system with a flow rate of 100 µL·min−1 and a constant temperature of 21 °C.

### 2.5. Cell Culture

The human non-small-cell lung cancer (H460), human prostate cancer (Du-145 and LNCaP), and human fetal lung fibroblasts (MRC-5) cell lines were purchased from the American Type Culture Collection (ATCC; Manassas, VA, USA). The human prostatic PNT1A cell line was kindly provided by Prof. Jędrzej Antosiewicz (Medical University of Gdańsk, Gdańsk, Poland). Cells were routinely tested for mycoplasma (Universal Mycoplasma Detection Kit—ATCC-30-1012 K; ATCC; Manassas, VA, USA) and found to be negative. H460, LNCaP, and PNT1A cells were cultured in RPMI 1640 medium (Sigma-Aldrich, USA). Du-145 and MRC-5 cells were grown in an EMEM medium (Eagle’s Minimal Essential Medium; Sigma-Aldrich, USA). Both media were supplemented with 10% FBS (fetal bovine serum; Biowest, Riverside, MO, USA), 100 µg·mL^−1^ of streptomycin (Sigma-Aldrich, Shanghai, China), and 100 unit·mL^−1^ of penicillin (Sigma-Aldrich, Rehovot, Israel). MRC-5 cells were cultured in an EMEM medium with 10% FBS without antibiotics. All cells were incubated in a humidified atmosphere containing 5% CO_2_ at 37 °C. Experiments were performed with cells in the exponential phase of growth.

### 2.6. The Used Concentration of Compounds in Experiments

The cytotoxic activity at IC_50_ and IC_80_ values of QD_green/red_, *β*-CD, QD_green/red_-*β*-CD-FA, C-2028, *β*-CD(C-2028), QD_green/red_-C-2028, and QD_green/red_-*β*-CD(C-2028)-FA nanoconjugates towards H460, Du-125, LNCaP, MRC-5, and PNT1A cells was examined previously by MTT assay [22]. All experiments were performed in the concentration which corresponded to the estimated IC_80_ value for C-2028 alone following 72 h of incubation (in the case of cancer cells). The used concentrations (IC_80_ value) in the experiments for C-2028, *β*-CD(C-2028), QD_green_-C-2028, and QD_green_-*β*-CD(C-2028)-FA nanoconjugates were 0.035 μM for H460 and MRC-5, 0.024 μM for Du145, and 0.133 μM for LNCaP and PNT1A cell lines, respectively. The concentration of QD_green_, *β*-CD, and QD_green_-CD-FA, corresponding to the IC_80_ value of unbound C-2028 compound in the nanoconjugates, was 0.0012 mg·mL^−1^ for H460 and MRC-5, 0.0008 mg·mL^−1^ for Du-145, and 0.0044 mg·mL^−1^ for LNCaP and PNT1A cell lines, respectively.

### 2.7. Confocal Microscopy Imaging

To explore the intracellular fate of compounds and their influence on lysosomal content in the cells, Confocal Laser Scanning Microscopy (CLSM) was used. Thanks to the fluorescence properties of QD_green_ and C-2028, they can serve as intrinsic fluorescence probes to explore their cellular uptake. Briefly, 1 × 10^6^ cells were seeded in the 60 mm plate with glass coverslips (except control, QD_green_, and QD_green_-*β*-CD-FA—2 × 10^5^ and 6 × 10^5^ cancer and normal cells were seeded, respectively) in 5 mL of medium and incubated overnight. Next, cells were treated with QD_green_, QD_green_-*β*-CD-FA, *β*-CD, C-2028, *β*-CD(C-2028), QD_green_-C-2028, and QD_green_-*β*-CD(C-2028)-FA nanoconjugates at IC_80_ values corresponding to C-2028 alone for 72 h. To explore the influence of compounds on lysosomal content in the cells, LysoTracker Red DND-99 (Invitrogen, Waltham, MA, USA) was added to a final concentration of 100 nM for 15 min at 37 °C. In immunofluorescence studies (F-actin) the cells were washed in PBS 1×, fixed with 3.7% paraformaldehyde (Sigma-Aldrich, USA) in PBS for 15 min at RT, and permeabilized in 0.1% Triton-X100 (Sigma-Aldrich, USA) in PBS for 15 min. Next, the cells were washed twice with PBS 1×, blocked with 2% bovine serum albumin (BSA; Sigma-Aldrich, USA) in PBS for 1 h at RT, and incubated for 50 min at 37 °C in a humidified chamber with the Alexa Fluor^®^ 647 phalloidin (1:250; Invitrogen, USA). Next, cells were washed twice with PBS 1× and immediately observed using the CLSM (63× magnification; ZEISS LSM 800, Oberkochen, Germany). In immunofluorescence studies, images were acquired additionally with an Airyscan detector using a × 63 1.4 NA Plan Apochromat objective (ZEISS LSM 800, Germany). The imaging conditions were: QD_green_ (*λ*_ex_ = 300 nm, *λ*_em_ = 543 nm), C-2028 (*λ*_ex_ = 528 nm, *λ*_em_ = 553 nm), Alexa Fluor^®^ 647 phalloidin (*λ*_ex_ = 650 nm, *λ*_em_ = 668 nm) and LysoTracker Red DND-99 (*λ*_ex_ = 578 nm, *λ*_em_ = 700 nm). The Mean Fluorescence Intensity (MFI) of LysoTracker Red DND-99 from the images of cells treated with QD_green_, QD_green_-*β*-CD-FA, *β*-CD, C-2028, *β*-CD(C-2028), QD_green_-C-2028, and QD_green_-*β*-CD(C-2028)-FA nanoconjugates, was performed with ImageJ software (version 1.8.0., Madison, WI, USA).

### 2.8. Preparation of Cell Lysates

The concentrations of C-2028 in the cell lysates were determined by the reversed-phase HPLC method. Human H460 (cancer) and MRC-5 (normal) lung cells were plated in 60 mm Petri dishes. After reaching the exponential phase of growth, the cells were treated with 35 μM unbound C-2028 and QD_green_-*β*-CD(C-2028)-FA nanoconjugate for 1, 12, and 24 h. At predetermined intervals of time, 1.0 mL of the cell culture medium was placed in an Eppendorf tube for further HPLC analysis, and the residue was discarded. The cells remaining on the Petri dish were collected with a scraper and washed once with PBS 1× (pH 7.4). Next, the cells were suspended in 400 μL of RIPA lysis buffer 1× (pH 7.5) (Abcam, Cambridge, UK) for 1 h on ice. Then, the mixture was centrifuged at 1400 rpm for 10 min at 4 °C. The resulting supernatants (cell extracts) were collected in a new Eppendorf tube and were directly analyzed by reversed-phase HPLC. Blank homogenate samples of the cells that were not treated with C-2028 and its nanoconjugate were also assayed.

The concentrations of C-2028 in the analyzed samples were calculated from the standard curve of the drug. A stock solution of C-2028 (100 μM) was prepared in water. The standard curve of C-2028 was established from ten standard calibration samples over the 0.25–100 μM concentration range and it was performed in triplicate. Peak areas of C-2028 vs. the corresponding drug concentrations were plotted and the obtained standard curve was: area under the C-2028 HPLC peak = 38,393.36 × C − 2028 concentration − 72,582.90 (*R*^2^ = 0.99).

### 2.9. HPLC Analysis of Cell Lysates

To receive concentration profiles of C-2028 in the selected cells treated with unbound C-2028 or its QD_green_-*β*-CD(C-2028)-FA nanoconjugate, the obtained cell lysates were analyzed directly by reversed-phase HPLC. Analysis was performed using Waters Associates HPLC system (Waters Co., Milford, MA, USA) consisting of a model 600E system controller, a model 7725i Rheodyne injector, 717 plus Autosampler, and a model 2996 photodiode array detector controlled with Empower 3 software (Waters Co., USA). Chromatographic separation was achieved using a reversed-phase Suplex pKb-100 C18 analytical column (4.6 × 150 mm, 5-µm particle size) (Supelco, Inc., Bellefonte, PA, USA). The mobile phase consisted of a mixture of (A) water with 0.1% formic acid, and (B) methanol with 5% water. 50 mL of each sample was injected into the HPLC column at a flow rate of 0.6 mL·min^−1^ with the following linear gradient elution: 15% B from 0–15 min, 100% B from 15–17 min, 100% B from 17–17.5, 15% B from 17.5–5 min (returning to initial conditions). The autosampler and the column were kept at 4 °C and RT, respectively. The eluate was monitored with UV-Vis detection at 420 nm and/or photodiode array and multiple wavelength detection.

### 2.10. Statistical Analysis

All experiments were carried out with at least three independent replicates and the results were presented as a mean ± standard deviation (SD). Statistical significance was determined using the RM one-way ANOVA with Dunnett’s multiple comparisons vs. C-2028. The two groups were considered statistically significant when * *p* < 0.05, ** *p* < 0.01. Pearson’s correlation coefficient of cells was calculated using ZEN System 2.6. software (ZEISS LSM 800, Oberkochen, Germany).

## 3. Results and Discussion

### 3.1. Degradation Pathway of QDs-β-CD(C-2028)-FA Nanoconjugates—In Vitro Studies

**Figure 2:**
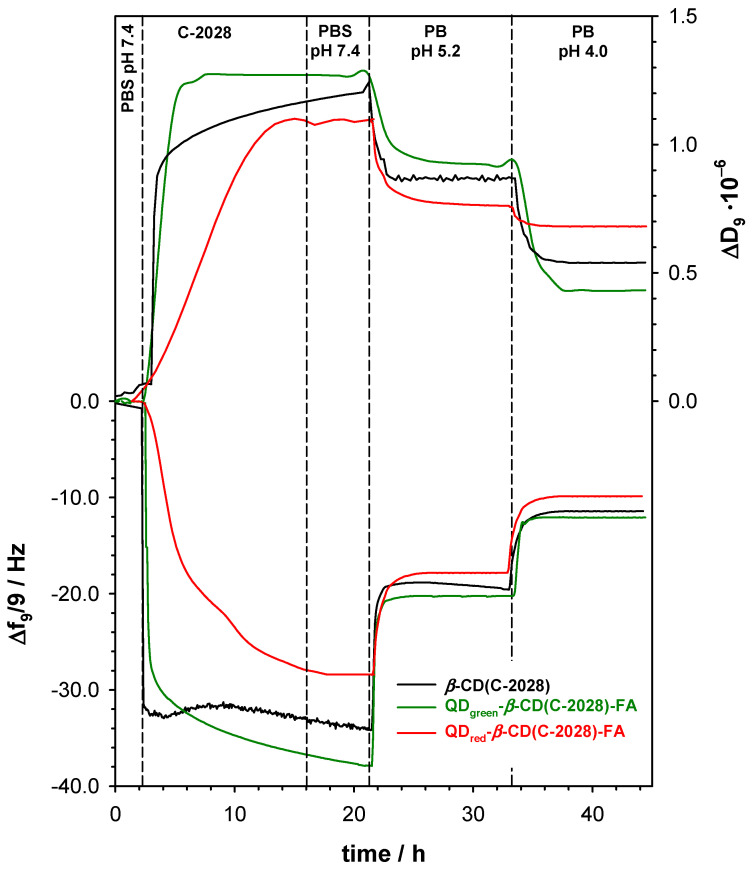
Typical QCM-D spectra of the shifts in frequency (Δ*f*) and dissipation factor (Δ*D*) recorded during the interaction of C-2028 with Au/QD-*β*-CD-FA (red and green lines) and Au/*β*-CD (black lines), and nanoconjugates degradation in various pH solutions. Experimental conditions: 0.02 M PBS (pH 7.4); C_QDs-*β*-CD-FA_ = 1.0 mg·mL^−1^; C_C-2028_ = 300 µM; C*_β_*_-CD_ = 1.0 mg·mL^−1^.

According to the current knowledge, the use of a selective self-navigating molecule (FA) should enhance the receptor-mediated endocytosis of the QDs-*β*-CD(C-2028)-FA nanoconjugates. The type of endocytosis process and macromolecule structure conduit a series of sorting steps in which the endosome is either transported to certain cell organelles, returns to the cell surface, or forms primary and secondary lysosomes. The rotation of the endosome in the cell depends on the pH value: pH 7.2–7.4 corresponds to extracellular space, pH 6.5–5.0 corresponds to endosomes, and pH 4.5–4.0 is characteristic of the environment in primary and secondary lysosomes [27]. According to the literature [28,29], there are two main mechanisms of the drug-carrier nanoconjugate degradation in the cancer cell called lysosomotropic and/or endosomotropic pathways. In our previous studies [22], we demonstrated that QD_green_-*β*-CD(C-2028)-FA nanoconjugates enter cells through multiple endocytosis pathways, including clathrin-mediated endocytosis (CME), caveolae-mediated endocytosis (CavME), and macropinocytosis (MP). To confirm the C-2028 loading into the QDs-*β*-CD-FA nanoconjugates and to get information about the drug release profiles, the QCM-D measurements were performed in the solutions with pH values: 7.4, 5.2, and 4.0 using the 3rd to 13th overtones. The typical frequency (Δ*f*) and the dissipation factor (Δ*D*) changes, for one selected overtone, during loading of the C-2028 compound inside the *β*-CD cavity are presented in Figure 2.

The modification step of the gold quartz crystal with QDs-*β*-CD-FA hybrids was performed outside of the QCM-D chamber by placing the droplet of 100 µL of QDs-*β*-CD-FA solution (1 mg·mL^−1^) on the Au surface and leaving it to dry in the desiccator. Then, the modified crystal (Au/QDs-*β*-CD-FA) was placed in the chamber and the cell was filled with 0.02 M PBS buffer at pH 7.4. After stabilization of the frequency and dissipation factor in the pure PBS buffer, the C-2028 compound in the concentration of 100 mM was added to the reaction chamber. The addition of an unsymmetrical bisacridine derivative to the buffer flowing through the chamber caused a decrease in the Δ*f* value and an increase in the Δ*D* value was observed. This effect was a consequence of the formation of an inclusion complex between *β*-CD and C-2028. The process of encapsulation of C-2028 inside the *β*-CD cavity finished after ca. 10 h; the frequency shift reached a maximum of −37.0 Hz and stopped change. The mass of C-2028 loaded into the QDs-*β*-CD-FA hybrids were 654.90 and 504.45 ng·cm^−2^ for QD_green_-*β*-CD-FA and QD_red_-*β*-CD-FA, respectively. To remove unbounded C-2028 molecules and get information about the stability of the formed inclusion complex in physiological conditions the solution in the chamber was changed to the pure 0.02 M PBS buffer (pH 7.4). The exchange of the solution caused some perturbations in the Δ*f* and Δ*D* values but only in the case of QD_green_-*β*-CD(C-2028)-FA nanoconjugate. The QD_green_ nanocrystals, contrary to the QD_red_ nanocrystals, contain two coordination spheres of ligands that stabilize zinc ions [19]. The ligands directly bound to the surface formed the first sphere, whereas the second coordination sphere is free ligands being in balance with the ligands of the first sphere. Two such coordination spheres are probably responsible for the layer motion. Moreover, the presence of two coordination spheres increases the surface area of the QD_green_. The larger surface must result in the number of cyclodextrin molecules conjugated with quantum dots and as consequence in the mass of the loaded drug. The decoration of *β*-CD molecules with selective self-navigating molecules (FA) can limit the efficiency of the formation inclusion of complex *β*-CD(C-2028). To verify this hypothesis, the control experiment using modified crystal Au/*β*-CD was carried out. The observed frequency and dissipation changes (black lines in Figure 2) were very similar to those recorded during the loading step of C-2028 into QDs-*β*-CD-FA hybrids. It confirmed that the unsymmetrical bisacridine derivative can form the inclusion complexes with the *β*-cyclodextrin present in the QDs-*β*-CD-FA hybrids.

**Figure 3:**
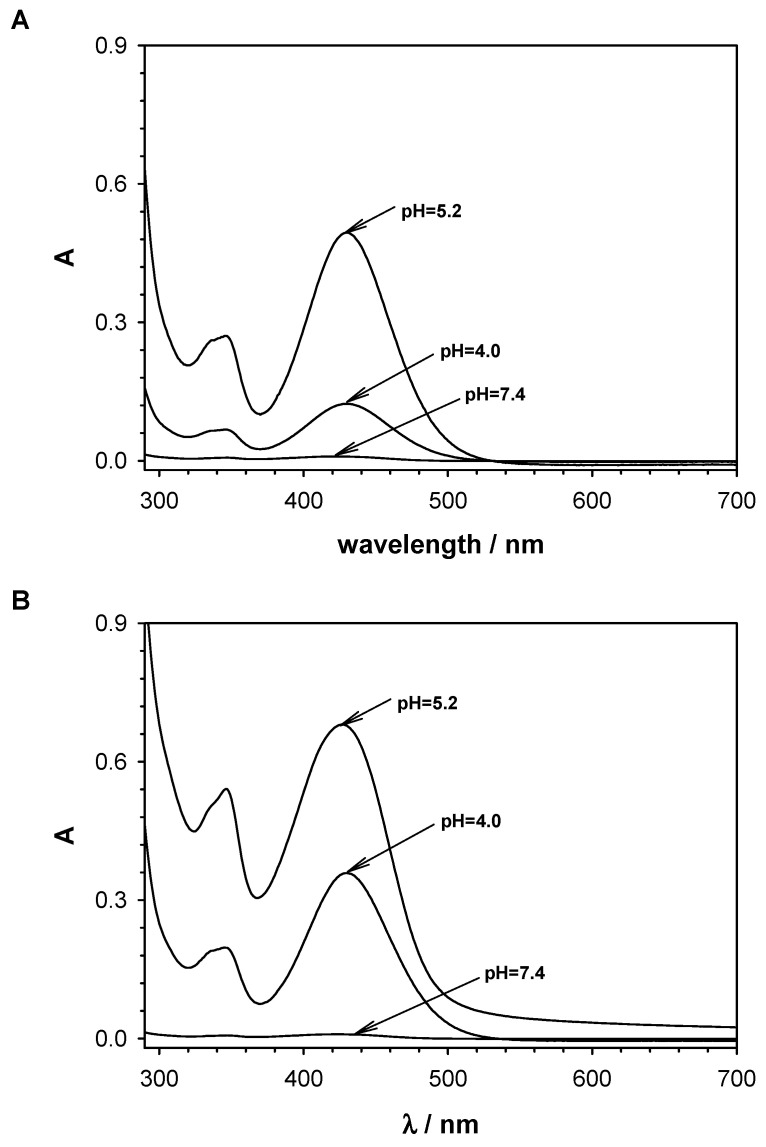
UV-Vis spectra of solutions obtained during releasing steps of compound C-2028 from the QD_red_-*β*-CD(C-2028)-FA (**A**), and QD_green_-*β*-CD(C-2028)-FA (**B**) nanoconjugates.

To demonstrate that C-2028 was released from the QDs-*β*-CD(C-2028)-FA nanoconjugates, the solutions were collected after each step of passing the appropriate pH buffer through the QCM-D chamber and analyzed by UV-Vis spectroscopy. The recorded UV-Vis spectra are presented in Figure 3. No absorbance band characteristic for the C-2028 compound at 430 nm after washing with PBS buffer pH 7.4 indicates the high stability of the inclusion complex *β*-CD(C-2028) under physiological conditions. This band appeared only after using a buffer with a lower pH. It means that the compound C-2028 was partially released from QDs-*β*-CD(C-2028)-FA nanoconjugates at both pH 5.2 and 4.0. The intensity of the absorbance bands at 430 nm was higher in the case of QD_green_-*β*-CD(C-2028)-FA nanoconjugate than for QD_red_-*β*-CD(C-2028)-FA nanoconjugate for both releasing buffers. This confirmed that the amount of C-2028 accumulated in QD_green_-*β*-CD(C-2028)-FA nanoconjugate is higher than in the case of QD_red_-*β*-CD(C-2028)-FA. Thus, the obtained UV-Vis results are in very good agreement with the QCM-D results and confirm that C-2028 is released from the nanoconjugate via both the endosomotropic and lysosomotropic pathways.

To define the degradation pathway of the QDs-*β*-CD(C-2028)-FA nanoconjugates the QCM-D experiments in two selected pH values (5.2 and 4.0) of the environment were performed. The change of PBS buffer in the reaction chamber from pH 7.4 to 5.2 led to the increase of the frequency of the quartz crystal to the value of ca. −20.2 Hz. The use of a buffer at pH 5.2, characteristic of the environment inside the endosome, partially removed ca. 48% of the compound C-2028 from the nanoconjugate. Further reduction of the pH to the value of 4.0, for the characteristic of the environment in the liposome, resulted in the release of another batch of C-2028 at the level of 18% to the introduced amount.

**Figure 4:**
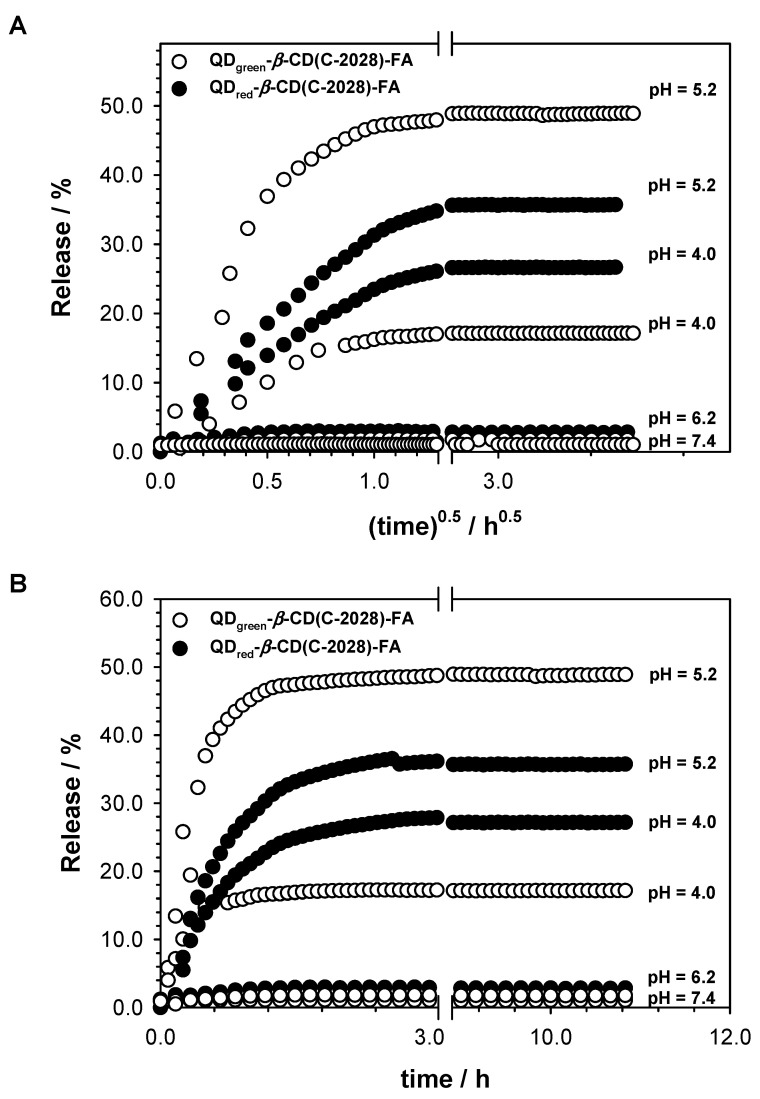
The in vitro release profiles of C-2028 from QDs-*β*-CD(C-2028)-FA nanoconjugates in four media differ in pH (pH: 7.2, 6.2, 5.2, and 4.0) according to Higuchi (**A**), and Korsmeyer–Peppas (**B**) models.

To get information about the mechanism of C-2028 releasing from the nanoconjugate, the release profiles were plotted as cumulative percentage drug release vs. square root of time and time, see Figure 4. It is worth emphasizing that the stability of the obtained QDs-*β*-CD(C-2028)-FA nanoconjugates was very good both in the tumor microenvironment (pH 6.2) and in the normal tissue environment (pH 7.4). Therefore, the simple Higuchi and Korsmeyer–Peppas models were applied to determine release parameters. The obtained data as a result of fitting experimental data to Equations (1) and (2) are presented in Table 1.

Due to the presence of the folic acid in the nanoconjugate structure, the drug in this form should enter the cell passing through the cell membrane mainly by endocytosis of the fluid phase with the participation of the receptor [28,29]. The individual phases of endocytosis are characterized by increasingly lower pH. In endosomes, the pH is reduced to the value of about 6.0–5.0, and after the endosome conjugation with lysosomes up to about 4.0. The pH shift could contribute to the release of the drug from the drug-carrier conjugate especially when the drug remains in the tumor cell for a longer period of time. The obtained data showed that the process of drug release takes place in the intracellular space through the lysomotropic endosomotropic pathways. Taking into account the value of the parameter *n,* the release mechanism of drugs is found to be Fickian diffusion, where the driving force is a concentration gradient of the releasing compound. Moreover, the intensity of drug release from the nanoconjugates is more effective at pH 5.2. It is worth stressing that lowering the pH of the environment to the value of 4.0 did not lead to the complete release of the compound. No significant release at pH 7.4 and significant release of the C-2028 in the acidic media from the QDs-*β*-CD(C-2028)-FA nanoconjugates suggests that one can expect controlled drug release in the cancer cells and no uncontrolled drug release in the bloodstream. It is well-known that cancer cells’ environments show acidic pH values (pH 4–6) [30,31]. The release process is rather slow (low value a *k_H_*), which is beneficial because it provides therapeutic concentrations of the drug substance at the selected site for a long time [32]. The release parameters obtained for the QDs-*β*-CD(C-2028)-FA nanoconjugates were very close to those obtained for the complex of C-2028 with *β*-cyclodextrin. Such behavior suggests that the compound is introduced to the nanoconjugate only through the formation of inclusion complexes with *β*-CD. Any interactions of the C-2028 compound with QD or FA did not take place.

The solution of the C-2028 compound is a colored solution, so UV-Vis spectroscopy was used to determine the stability constants of the formed complexes as a function of pH using Benesi–Hildebrand dependence [33]. Upon the addition of *β*-CD to the C-2028 solution, a decrease in the absorbance at 425 nm was observed (Figure S1 in Supplementary Materials). This decrease was significantly greater in the situation of solutions with pH close to physiological pH. The values of the formation constants determined for the *β*-CD(C-2028) complex are: 1.90 × 10^5^; 0.89 × 10^5^; 2.61 × 10^3^, and 5.72 × 10^2^ M^−1^ at pH 7.4, 6.2, 5.2, and 4.0, respectively. At pH 7.4, corresponding to the pH of body fluids, the stability constants of the complex are higher than at pH 5.2 or pH 4.0. The protonation of the nitrogen atoms in the acridine ring and in the alkyl linker between acridine and imidazoacridine rings changes the electron density distribution in C-2028, which can affect the *β*-CD-C-2028 interaction.

### 3.2. Cellular Fate of QDs-β-CD(C-2028)-FA Nanoconjugates and Their Influence on the Lysosomes Content in Cells

**Figure 5:**
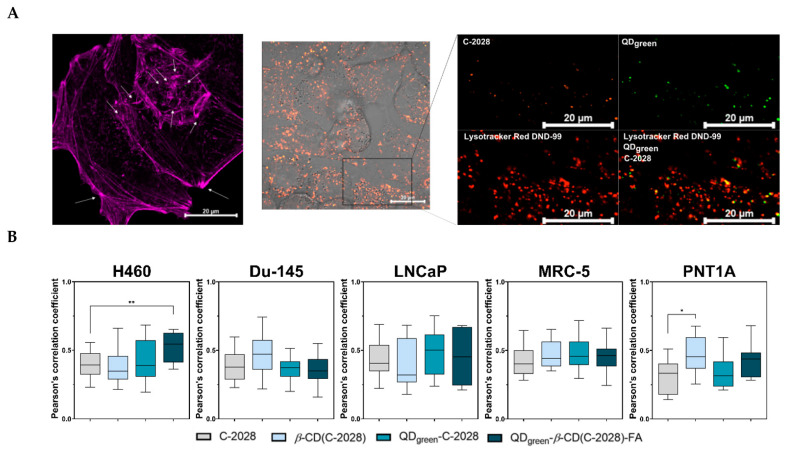
(**A**) Confocal images presenting localization of QD_green_-*β*-CD(C-2028)-FA in H460 cancer cells following 72 h of treatment. Acidic organelles are stained with Lysotracker Red DND-99 (red), F-actin in violet. C-2028 and QD_green_ are depicted in orange and green, respectively. The arrows indicate signals from C-2028 and QD_green_. The scale bar is 20 μm. (**B**) Pearson’s correlation coefficient of colocalization of unbound C-2028 and its nanoconjugates (*β*-CD(C-2028), QD_green_-C-2028, and QD_green_-*β*-CD(C-2028)-FA) with acidic organelles in H460, Du-145, LNCaP, MRC-5, and PNT1A cells. Data are expressed as the mean ± standard deviation, *n* = 27. The results were analyzed by one-way ANOVA with Dunnett’s multiple comparisons vs. unbound C-2028, * *p* < 0.05, ** *p* < 0.01.

The therapeutic efficiency of chemotherapeutic agents against cancer depends on the ability to reach their final targets. The cellular fate of most anticancer agents at the subcellular level is usually biological macromolecules, which play a key role in carcinogenesis [34]. Thanks to the combination of design, and specific modification, the subcellular targeting of nanoconjugates is enriched in cancer cells. These nanoconjugates can be internalized by endocytosis (e.g., lysosomal degradation) and target-specific subcellular structures [35]. Next, the controlled release of chemotherapeutic agents at the target sides can improve their anticancer efficiency and reduce the toxic effect [36]. In our previous studies [22], we demonstrated the use of FA as a targeting molecule, especially in QD_green_-C-2028 nanoconjugates (QD_green_-*β*-CD(C-2028)-FA), significantly increased the amount of C-2028 compound in lung H460 and prostate Du-145 cancer cells. Moreover, we showed that these nanoconjugates enter cells through endocytosis which takes place through several distinct pathways including clathrin-mediated endocytosis, caveolae-mediated endocytosis, and macropinocytosis. Therefore, we selected for further experiments nanoconjugates with QD_green_. The next step of our study was to explore the intracellular fate of compounds and their influence on lysosomal content in the cells. Thanks to the fluorescence properties of QD_green_ and C-2028, they can serve as intrinsic fluorescence probes to explore their cellular uptake and intracellular fate. To measure the influence of compounds on lysosomal content in the cells, LysoTracker Red DND-99 was used. This fluorescent marker selectively accumulates in acidic compartments, especially in late endosomes and lysosomes [37]. All of the studied compounds, after their internalization into the cells, were delivered to acidic organelles. In Figure 5A the localization of QD_green_-*β*-CD(C-2028)-FA nanoconjugate was presented. Orange and green signals are representative of C-2028 and QD_green_, respectively. The signals from these compounds covered signals from lysosomes loaded with LysoTracker Red DND-99 (red). In addition, Zen 2.6. software (ZEISS LSM 800, Germany) was used to calculate Pearson’s correlation coefficient (PCC) of colocalization of unbound C-2028 and its nanoconjugates (*β*-CD(C-2028), QD_green_-C-2028, and QD_green_-*β*-CD(C-2028)-FA) with acidic organelles, as shown in Figure 5B. The PCC was the highest in the case of H460 cells treated with QD_green_-*β*-CD(C-2028)-FA nanoconjugates (0.522). This meant that nearly 52% of these internalized nanoconjugates are still accumulated in lysosomes after 72 h of incubation. A similar result of the cellular fate of HAp-PEI/siKras was observed by Luo D. et al. [38]. They showed that signals from LysoTracker Red DND-99 and siRNA-FAM overlapped, indicating that HAp-PEI has successfully entered into the lysosomes. Furthermore, they can find that cargo effectively knocked down the expression of the *KRAS* gene and downregulated the expression of the Kras protein. Zeng Y. et al. showed that PPG-FA/Ce6 (folic acid (FA)-conjugated polyethyleneimine-modified PEGylated nanographene) was localized in the lysosomes after their internalization [39]. This dual delivery of siRNA and doxorubicin platforms (PPG-FA/siRNA/Dox) exhibited a gene-silencing effect and efficient intracellular delivery of Dox.

**Figure 6:**
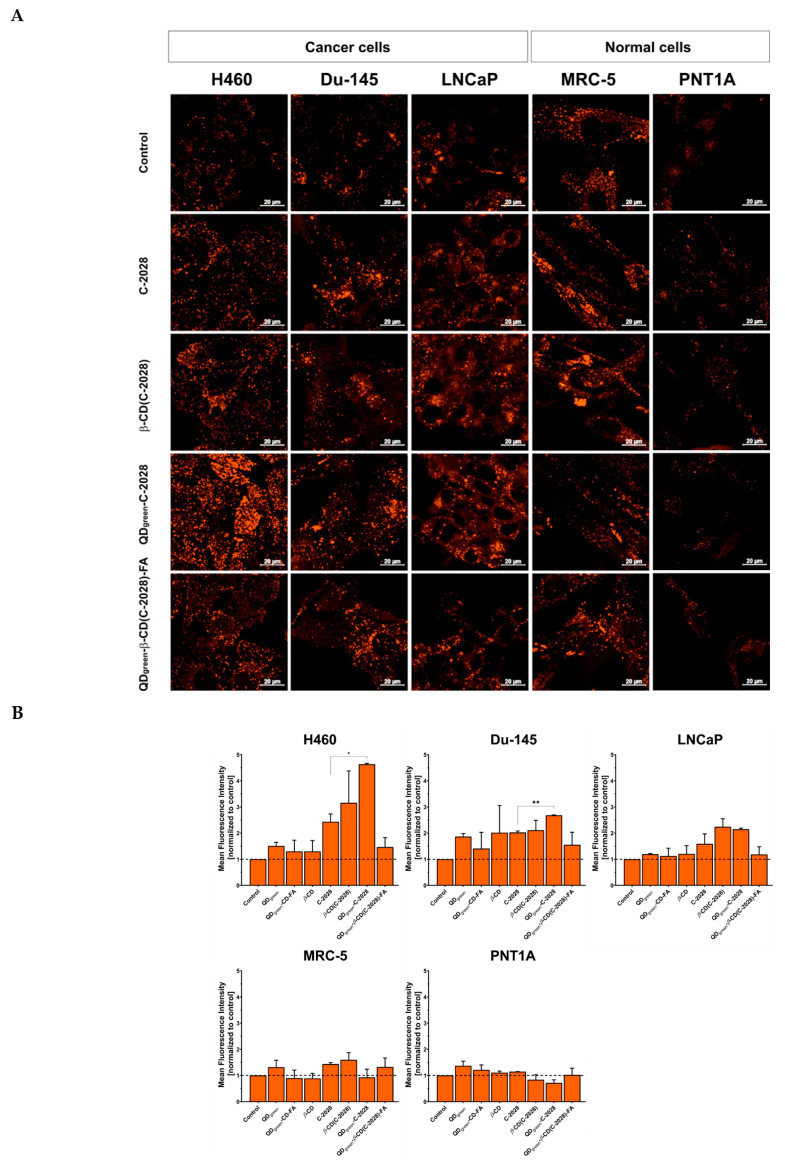
Intracellular fluorescence images of acidic organelles loaded with LysoTracker Red DND-99 (**A**), and their quantitative analysis of Mean Fluorescence Intensity (MFI) with ImageJ software (**B**) in H460, Du-145, LNCaP, MRC-5, and PNT1A cells incubated with QD_green_, QD_green_-*β*-CD-FA, *β*-CD, C-2028, *β*-CD(C-2028), QD_green_-C-2028, and QD_green_-*β*-CD(C-2028)-FA for 72 h. The scale bar is 20 μm. MFI values of C-2028 and its nanoconjugates were determined using ImageJ software. Data are expressed as the mean ± standard deviation of three independent experiments. The results were analyzed by one-way ANOVA with Dunnett’s multiple comparisons vs. unbound C-2028, * *p* < 0.05, ** *p* < 0.01.

The difficulty of controlled drug release at an intracellular level remains a key challenge for maximizing drug safety and efficacy. The degradation pathway of the nanoconjugates study shows that C-2028 was released from the QDs-*β*-CD(C-2028)-FA at both pH 5.2 and 4.0, corresponding to endosomes and lysosomes, respectively [27]. At the same time, no significant release of C-2028 from nanoconjugates was observed (Figure 2, Figure 3 and Figure 4). As we showed, all of the studied compounds were delivered to acidic organelles (Figure 5A). However, the Mean Fluorescence Intensity (MFI) of signals from lysosomes loaded with LysoTracker Red DND-99 was different, depending on the cell line as well as the used variant of the compound (Figure 6). The strongest difference between C-2028 and its nanoconjugates was observed in the case of lung H460 cancer cells. In this case, C-2028 alone induces the strongest signals from acidic organelles too. A significant difference between the MFI of signals for C-2028 and its nanoconjugates was observed for QD_green_-C-2028 in H460 and Du-145 cancer cells. The use of FA in nanoconjugates decreased the MFI of signals from lysosomes in cancer and normal cells. What is important, C-2028 alone and its nanoconjugates induced a stronger response (stronger MFI of signals from acidic organelles) in cancer cells, compared to normal cells. In normal MRC-5 and PNT1A cell lines, no significant changes in MFI of signals from lysosomes, following treatment with all studied compounds (QD_green_, QD_green_-*β*-CD-FA, *β*-CD, C-2028, *β*-CD(C-2028), QD_green_-C-2028, and QD_green_-*β*-CD(C-2028)-FA nanoconjugates) was observed (Figure S2 in Supplementary Materials). In all of the studied cell lines, there was no obvious difference between the MFI of signals from acidic organelles of control cells and platforms of the C-2028 compound (QD_green_, QD_green_-*β*-CD-FA, *β*-CD), except the Du-145 cell line for QD_green_ and *β*-CD.

### 3.3. HPLC Monitoring of C-2028 in H460 and MRC-5 Human Lung Cells Treated with the Unbound Drug and Its Nanoconjugate

**Figure 7:**
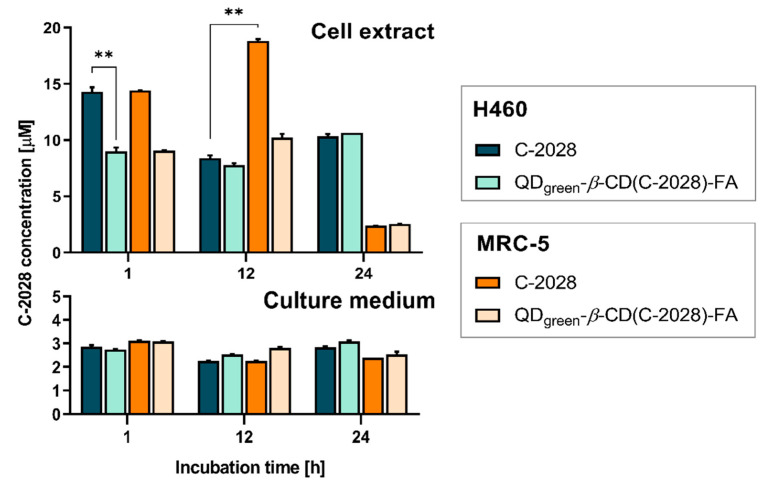
The concentration profiles of C-2028 in H460 (cancer) and MRC-5 (normal) lung cells treated with 35 μM unbound C-2028 and QD_green_-*β*-CD(C-2028)-FA nanoconjugate for 1, 12, and 24 h obtained by reversed-phase HPLC analysis of cell extract (**top**) and culture medium (**bottom**). Data are expressed as the mean ± standard deviation of two independent experiments. The results were analyzed by one-way ANOVA with Dunnett’s multiple comparisons vs. unbound C-2028 (in H460 cells), ** *p* < 0.01.

To obtain the desired therapeutic response in the target site, the nanoencapsulated drug must be released from its nanoconjugate. Distinguishing the encapsulated and the released drug is often problematic and there are technical difficulties in quantifying them in the complex matrices [40]. Many methods reported in the literature for assaying the release profiles of drugs from nanoparticulate systems are based on HPLC with UV-Vis and/or mass spectrometric detection [41,42,43]. This approach may also be useful for the initial evaluation of the drug’s ability to penetrate cells. In the present work, we applied a traditional reversed-phase HPLC method (described in the Section 2) for the rapid determination of C-2028 content in cells treated with the unbound C-2028 and QD_green_-*β*-CD(C-2028)-FA nanoconjugate. The total drug concentrations in the samples collected (cell extracts and culture media) were measured as a function of incubation time (Figure 7). Among the studied cell lines, for this experiment, we chose H460 (cancer) and MRC-5 (normal) lung cells that showed the strongest differences in the MFI of signals from lysosomes. The C-2028 alone and its nanoconjugate (QD_green_-*β*-CD(C-2028)-FA) induced a stronger response (stronger MFI of signals from acidic organelles) in H460 cancer cells, compared to normal MRC-5 cells. Moreover, the H460 cells also expressed higher sensitivity to the action of the unsymmetrical bisacridine than MRC-5 cells.

From the graphs in Figure 7, it is clearly seen that C-2028 entered both H460 and MRC-5 cells easily within the first hour of incubation. This observation complies with our previous work, where we showed kinetic cellular uptake of C-2028 and its nanoconjugates to cancer and normal cells using CLSM [22]. While in the culture media, it remained at a constant and relatively low concentration (*ca.* 2.2–3 μM) regardless of the time of incubation, another tendency was observed in the cell extracts. The increase in incubation time up to 12 h resulted in the decrease of the C-2028 amount only in H460 cells, with a release of ca. 8.4 μM and 7.8 μM in unbound C-2028 and its nanoconjugate, respectively. In turn, we noticed a clear increase in the C-2028 concentration of the unbound drug in MRC-5 cells, however, drug release was still slightly slow in these cells treated with QD_green_-*β*-CD(C-2028)-FA nanoconjugate. In contrast, more C-2028 was revealed in cancer cells after 24 h and C-2028 encapsulated in nanoconjugate penetrated H460 cells comparable to unbound C-2028. The intracellular concentration of C-2028 in normal MRC-5 cells dropped rapidly after this time, which may indicate that the drug was probably pumped out of the cells. Moreover, the pH of acidic organelles promoted an increased release of C-2028 from its nanoconjugates. Since pH in normal cells is higher than in cancer cells [31], the release of C-2028 from its nanoconjugates is decreased, especially for a longer incubation time (24 h). This can be responsible for weaker biological responses in the case of normal cells, and at the same time, stronger responses in the case of cancer cells. Overall, our results supported the interpretation of the results from our previous studies [22] by confirming that the use of FA as a cancer-targeting molecule indeed improves the entry of the drug into cancer cells. We can predict that by extending the incubation time of cancer cells with a nanoconjugate, a greater amount of C-2028 could successfully diffuse out from the nanoparticle in the target cancerous site and thus be able to remain there for a longer time.

## 4. Conclusions

Traditional small-molecule chemotherapeutics are often delivered without any specific molecular-targeting strategy. However, to optimize their potential benefits for patients, there is a need for advanced drug delivery systems for the targeted and controlled release of biologically active molecules in tissues and cells. It is known that the presence of a self-navigating molecule (e.g., folic acid, transferrin) favors the targeted delivery of drugs to tumor tissue through mediated endocytosis. The quantum dots decorated with *β*-cyclodextrin served as a carrier for C-2028 (potential anticancer drug), whilst the folic acid facilitates drug delivery to tumor tissue. The synthesized nanoconjugate QD_green_-*β*-CD-FA was fully physiochemically characterized in our previous paper [22]. Moreover, we showed that the use of FA in the nanoconjugates (QD_green_-*β*-CD(C-2028)-FA) significantly improved the cellular uptake of C-2028, especially in cancer H460 and Du-145 cells. Importantly, this effect was stronger in tumors (H460 and Du-145) than in normal cells (MRC-5 and PNT1A). In the present work, we demonstrated that both unbound and nanoencapsulated C-2028, after their internalization into cells, were delivered to acidic organelles (i.e., endosomes and lysosomes; Figure 6) present in abundance in cancer cells. Moreover, their pH promoted an increased release of C-2028 from its nanoconjugates. Under physiological conditions, pH 7.4, the QD_red/green_-*β*-CD(C-2028)-FA nanoconjugates are stable, as elucidated by QCM-D studies as well as dynamic scattering and zeta potential assays. The degradation of studied nanoconjugates occurs in acidic conditions. Although the drugs were introduced to the nanocarrier via weak interactions (inclusion complex), only ca. 60% of loaded C-2028 was released, of which about 50% (QD_green_-*β*-CD(C-2028)-FA) and 34% (QD_red_-*β*-CD(C-2028)-FA) during pH 5.2, remaining parts 14% and 26%, respectively, from nanoconjugates with QD_green_ and QD_red_ at pH 4.0. The UV-Vis analyses of eluates proved that the C-2028 is liberated much more easily at pH 5.2. The concentration profiles of C-2028 in the selected cells treated with unbound C-2028 or its QD_green_-*β*-CD(C-2028)-FA nanoconjugate were obtained from analysis of the cell lysates using reversed-phase HPLC. QCM-D measurements and the results of the HPLC analyses confirmed the findings obtained at an intracellular level. To conclude, based on the overall results, the novelty of our studies lies in the demonstration that QD_green_-*β*-CD-FA nanoplatforms can be used as traceable and pH-responsive drug delivery systems. Future work will focus on the influence of the use of these nanoplatforms on biological responses in cancer and normal cells, including cell cycle analysis and induction of apoptosis. We also plan to perform experiments with in vivo models or other platforms that mimic in vivo systems to check the performance of the proposed drug delivery system in a physiological environment.

## Figures and Tables

**Table 1:** Change in coefficient regression (*r*), release rate constant (*k_H_*), and release exponent (*n*) for the studied QDs-*β*-CD(C-2028)-FA nanoconjugates.

Media	Higuchi Model		Korsmeyer–Peppas Model	
	*k_H_* [µg × h^−0.5^]	*r^2^*	*n*	*r^2^*
**QD_green_-*β*-CD**(**C-2028**)**-FA**				
pH = 7.4	1.41	0.994	0.0029	0.990
pH = 6.2	2.62	0.993	0.099	0.989
pH = 5.2	70.7	0.991	0.101	0.987
pH = 4.0	20.3	0.992	0.074	0.992
**QD_red_-*β*-CD**(**C-2028**)**-FA**				
pH = 7.4	1.46	0.991	0.0034	0.988
pH = 6.2	5.09	0.989	0.101	0.993
pH = 5.2	36.5	0.987	0.156	0.992
pH = 4.0	25.6	0.986	0.160	0.991
***β*-CD**(**C-2028**)				
pH = 7.4	2.41	0.990	0.016	0.993
pH = 6.2	4.75	0.989	0.0078	0.988
pH = 5.2	65.2	0.995	0.213	0.991
pH = 4.0	19.4	0.993	0.104	0.986

## Data Availability

Data are contained within the article, Supplementary Materials, and Most Wiedzy Portal. The DOI number of raw data in Most Wiedzy Portal is https://doi.org/10.34808/a2v2-c880.

## Supplementary Materials

The following supporting information can be downloaded at: https://www.mdpi.com/article/10.3390/pharmaceutics15010201/s1, Figure S1: UV-Vis titration curves obtained for *β*-CD(C-2028) complex in various pH: 7.4 (A), 6.2 (B), 5.2 (C), and 4.0 (D). Figure S2: Intracellular fluorescence images of acidic organelles loaded with LysoTracker Red DND-99 in H460, Du-145, LNCaP, MRC-5, and PNT1A cells incubated with QD_green_, QD_green_-*β*-CD-FA, *β*-CD for 72 h. The scale bar is 20 μm.
