# Supplementary material for: pH-Responsive Drug Delivery Nanoplatforms as Smart Carriers of Unsymmetrical Bisacridines for Targeted Cancer Therapy
Source: Pharmaceutics. 2023 Jan 6;15(1):201. doi: 10.3390/pharmaceutics15010201 (PMC9861370; doi:10.3390/pharmaceutics15010201)
Supplement: Supplementary file 1 [file pharmaceutics-15-00201-s001.zip › pharmaceutics-2089116-supplementary.pdf]

Supplementary Materials

pH-Responsive Drug Delivery Nanoplatforms as Smart Carriers of Unsymmetrical Bisacridines for Targeted Cancer Therapy

Joanna Pilch [1,*], Agnieszka Potęga [1], Agata Kowalczyk [2], Artur Kasprzak [3], Patrycja Kowalik [2,3], Piotr Bujak [3], Ewa Paluszkiewicz [1], Ewa Augustin [1] and Anna M. Nowicka [2,*]

[1] Faculty of Chemistry, Gdańsk University of Technology, Narutowicza Street 11/12, 80-233 Gdańsk, Poland
[2] Faculty of Chemistry, University of Warsaw, Pasteura Street 1, 02-093 Warsaw, Poland
[3] Faculty of Chemistry, Warsaw University of Technology, Noakowskiego Street 3, 00-664 Warsaw, Poland
* Correspondence: joanna.pilch@pg.edu.pl (J.P.); anowicka@chem.uw.edu.pl (A.M.N.)

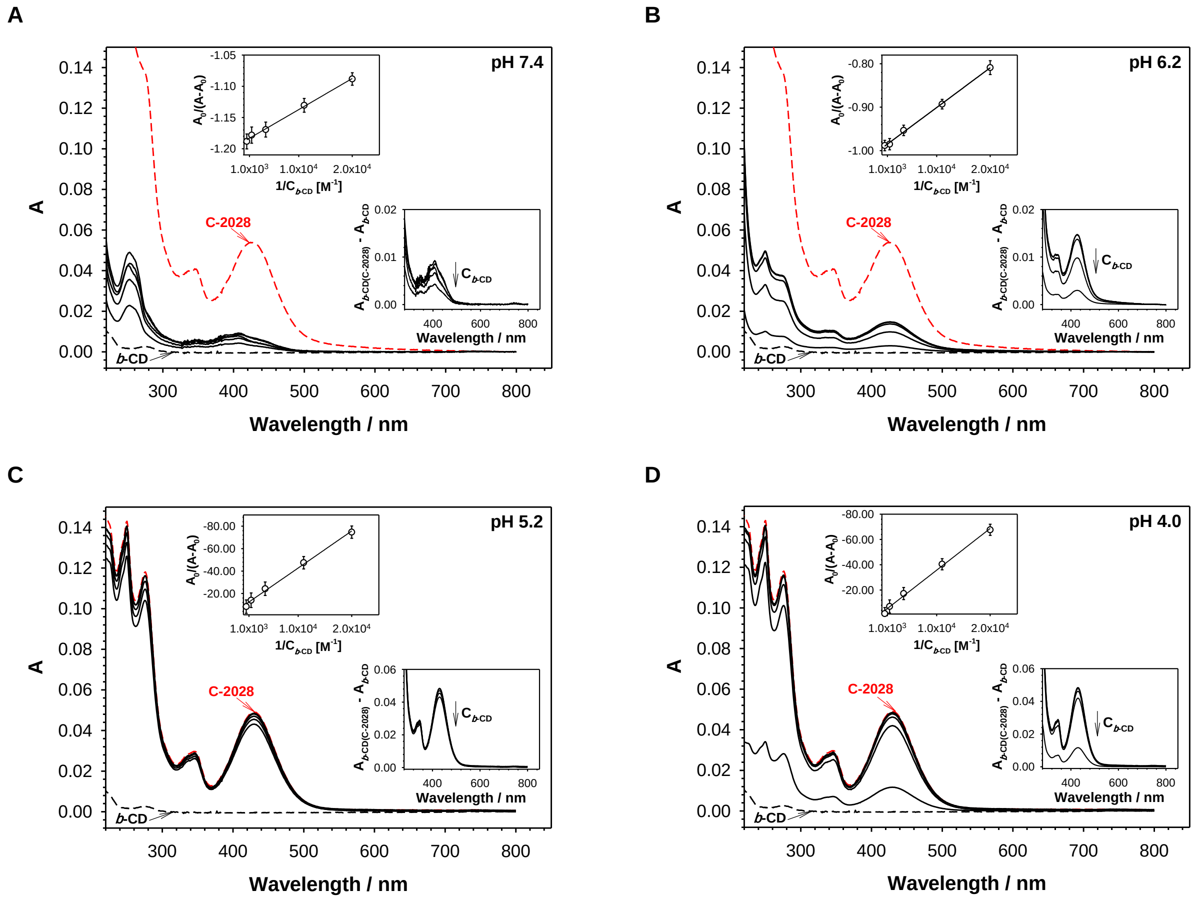

Figure S1. UV-Vis titration curves obtained for β−CD(C−2028) complex in various pH: 7.4 (**A**), 6.2 (**B**), 5.2 (**C**), and 4.0 (**D**).

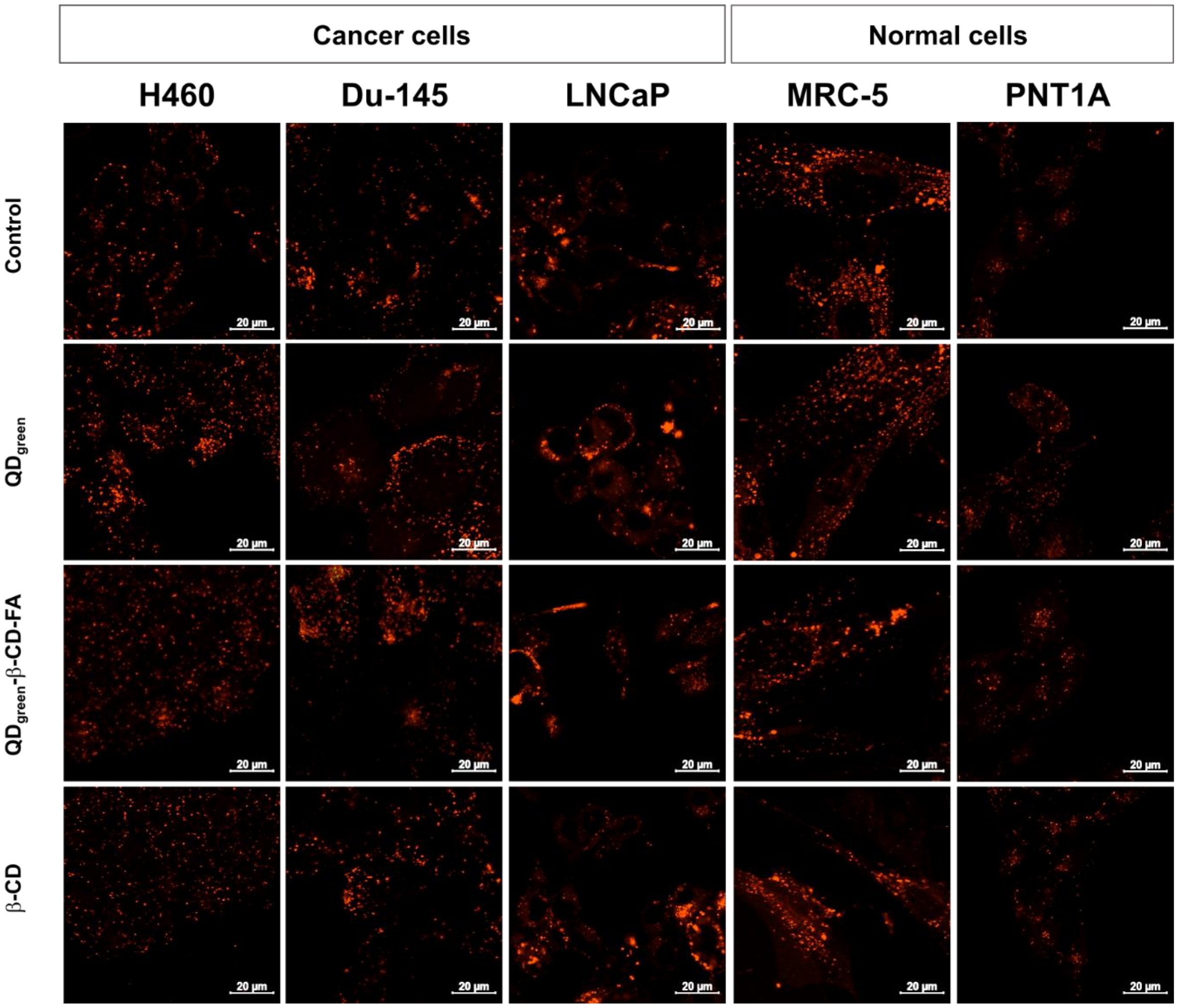

Figure S2. Intracellular fluorescence images of acidic organelles loaded with LysoTracker Red DND-99 in H460, Du-145, LNCaP, MRC-5, and PNT1A cells incubated with QD_{green}, $QD_{green}-\beta-CD-FA$, $\beta-CD$ for 72 h. The scale bar is 20 μm.
